# Gathering data for decisions: best practice use of primary care electronic records for research

**DOI:** 10.5694/mja2.50026

**Published:** 2019-03-30

**Authors:** Rachel Canaway, Douglas IR Boyle, Jo‐Anne E Manski‐Nankervis, Jessica Bell, Jane S Hocking, Ken Clarke, Malcolm Clark, Jane M Gunn, Jon D Emery

**Affiliations:** ^1^ Department of General Practice University of Melbourne Melbourne VIC; ^2^ Health and Biomedical Informatics Centre University of Melbourne Melbourne VIC; ^3^ Melbourne Law School University of Melbourne Melbourne VIC; ^4^ School of Population and Global Health University of Melbourne Melbourne VIC; ^5^ Networked Society Institute University of Melbourne Melbourne VIC

**Keywords:** Population health, General practice, Health services research, Data collection, Technology, Evidence‐based medicine, Ethics, research, Datasets as topic

## Abstract

- In Australia, there is limited use of primary health care data for research and for data linkage between health care settings. This puts Australia behind many developed countries. In addition, without use of primary health care data for research, knowledge about patients’ journeys through the health care system is limited.
- There is growing momentum to establish “big data” repositories of primary care clinical data to enable data linkage, primary care and population health research, and quality assurance activities. However, little research has been conducted on the general public's and practitioners’ concerns about secondary use of electronic health records in Australia.
- International studies have identified barriers to use of general practice patient records for research. These include legal, technical, ethical, social and resource‐related issues. Examples include concerns about privacy protection, data security, data custodians and the motives for collecting data, as well as a lack of incentives for general practitioners to share data.
- Addressing barriers may help define good practices for appropriate use of health data for research. Any model for general practice data sharing for research should be underpinned by transparency and a strong legal, ethical, governance and data security framework.
- Mechanisms to collect electronic medical records in ethical, secure and privacy‐controlled ways are available.
- Before the potential benefits of health‐related data research can be realised, Australians should be well informed of the risks and benefits so that the necessary social licence can be generated to support such endeavours.

Making more effective use of data is part of a global movement to improve health information exchange, decision making and policy development, consumer and business outcomes, and development of products and services. However, Australia is falling behind.^1^ Australia's health sector reportedly “stands out among other developed countries as one where health information is poorly used”.^1^ Secondary use of electronic medical records (EMRs) for research purposes occurs throughout the world. It has the potential to provide significant public health gains by informing evidence‐based health care education, policy, practice and service delivery.^2^, ^3^, ^4^ But such use of EMRs in Australia is ad hoc. Also, non‐use of such data could have negative effects on the public, such as causing a financial burden on society.^1^, ^5^, ^6^

Combined data from primary care EMRs can be used to evaluate the outcomes of interventions, provide practitioners with evidence for clinical decision making, assess uptake of best practice principles, facilitate quality improvement, highlight inequities in access and outcomes, determine need for services, and potentially assist in earlier detection of disease.^3^, ^5^, ^7^ The scope of interdisciplinary research using primary care clinical datasets is enormous (Box 1). Further, the ability to link different data sources together (eg, primary care and hospital data) also has enormous value. It can increase the range of questions that research can answer, improve statistical properties of data, and improve use of resources.^5^, ^9^, ^10^, ^11^, ^12^ Despite these benefits, the full potential of such data‐based research has not been realised. In this article, we examine issues relating to the use of EMRs for research in Australia, and discuss how data extraction software can enable fuller use of EMRs in research, auditing, and surveillance of population health and disease. We also provide a model that shows how harnessing the untapped potential of EMRs can support decision making by general practitioners and thereby improve patient care.

**1 Types of research, research activities and advances in clinical care made possible with primary care clinical data sharing^3^, ^8^**

- Longitudinal cohort studies
- Data‐based research combined with interventional studies to assess outcomes of interventions such as new practices, medications, decision support tools and clinical trials
- Comparative effectiveness research to identify more clinically relevant and cost‐effective ways to diagnose and treat patients
- Research that identifies service needs and care inequalities
- Collection of data for randomised controlled trials and measuring outcomes
- Examination of primary health care use and billing to inform economic evaluations and health services research
- Evidence‐based identification of unnecessary repeat laboratory testing
- Data quality studies to inform data quality improvement programs
- Big data analytics of combined datasets to match treatments with outcomes and predict patients at risk of disease or hospital re‐admissions
- Improved matching of treatments to individual patients
- Predictive modelling to identify individuals at risk of developing a specific disease or who would benefit from preventive care
- Analytics to enable targeted educational interventions (for the public and general practitioners)

## Australian context

Australian general practices were early adopters of clinical practice software tools and EMRs.^13^ First‐generation general practice software assisted clinicians with drug prescribing, but over time evolved into many clinical patient management software packages. These packages were designed to help GPs manage patient care and referrals, and improve practice efficiency. However, each package has been developed with limited need to comply with clinical coding, interoperability, or national accreditation standards.^14^ Because of these limitations, little research and data linkage using EMRs has been conducted in Australia.

The first Australian primary care data linkage project started in Western Australia in 2007, when Medicare Benefits Schedule (MBS) and Pharmaceutical Benefits Scheme (PBS) data were linked to several state health care datasets. This enabled studies of the effects of primary care on hospitalisations and mortality for several chronic diseases.^9^, ^10^ However, the limited clinical information within MBS and PBS datasets meant that assumptions had to be made to elicit meaning from the data.

In 2012, the Australian Government introduced its digital health record system: My Health Record. As a result of the move from opt‐in to opt‐out in 2019, most Australians will soon have a My Health Record containing online summaries of their health information, unless they opt out (https://www.myhealthrecord.gov.au/). As outlined in the Australian Government's *Framework to guide the secondary use of My Health Record system data*, secondary use must be demonstrably consistent with “research and public health purposes” and “likely to generate public health benefits and/or be in the public interest.”^8^

Research on Australians’ opinions regarding appropriate secondary use of EMRs is very limited. Opinion polling of a nationally representative sample of 1011 Australians has indicated that there is strong support for the use of health records for research, with 93% of Australians either strongly or somewhat supporting it and only 7% opposed, and strong public trust in medical researchers (67% high or very high trust, 29% moderate trust).^15^ Most of those polled (84%) believed that health providers involved in research give the best care because they are more aware of new developments and the latest practices.^15^

Despite these positive opinions, researchers sometimes wait years for approvals to access patient data for research.^1^ Funding to access datasets is also an area of concern, with a recent news item suggesting that general practice research in Australia is nearing crisis point, largely due to inadequate funding, and not because of lack of GP enthusiasm for research.^16^ So the barriers to using EMRs for research or other secondary purposes need to be addressed.

## International experience

Internationally, public opinion on the appropriate use of EMRs for purposes other than providing direct clinical care is mixed.^17^ A systematic review of public opinion on the use of patient data for research in the UK and the Republic of Ireland was undertaken after the 2013 introduction, public backlash and 2016 closure of NHS England's care.data program. The care.data program enabled extraction of identifiable patient data from general practice records to the Health and Social Care Information Centre (now NHS Digital), where it was linked to other data sources (eg, hospital records) with plans to make linked, pseudonymised data available to a range of data recipients. The public backlash voiced concerns about privacy and security, sale of data to companies that could use it to generate profit, and lack of informed consent relating to use of identifiable patient records.^18^, ^19^ The project lacked the necessary social licence to proceed, which resulted in a loss of trust among GPs, patients and the public. Ultimately, this led to the downfall of the project, scepticism and closer scrutiny of future ventures of a similar nature.^20^, ^21^ The reviewers found that although consumers generally had little knowledge about secondary uses of data from EMRs, when it was explained, many were willing to share their data for the “common good” subject to safeguards.^17^ In New Zealand, public opinion has been found to be similar.^22^ Overall, public willingness to share data is qualified by concerns about data de‐identification and privacy, issues of trust (or distrust) relating to who can access the data, the amount of transparency regarding secondary use, security controls, and the ability to retain control over who can access data and for what purpose.^5^, ^17^, ^23^ Emerging from the research are best practice principles for the appropriate use of health data for research (Box 2).

**2 Best practice principles for appropriate use of health data for research^4^, ^17^, ^23^, ^24^, ^25^, ^26^**

- Data should be de‐identified on extraction (for privacy protection)
- If data are not de‐identified, informed patient consent should be obtained
- Data use or handling for private interests and financial gain is often objectionable (eg, use of data by health insurance companies for commercial profit)
- Independent data governance committees should decide who can access data
- Gain public trust around:
  ▶ an organisation's motivations for collecting and using data
  ▶ an organisation's competence in safeguarding data from hacking, unintentional data leakage, unauthorised access and data breach events
- Robust data security systems are needed, to provide data access only to trusted and approved users
- Data provided must be limited to the minimum required to answer the research question(s)
- Transparency must be evident at every stage and level of data use
- Community involvement is helpful in terms of fully realising the public benefits of data‐based health research
- Introducing dynamic consent approaches is beneficial — for example, approaches that move away from static, one‐off consent and move towards enabling individuals to exercise preferences (ie, who can access their data and what their data can be used for) over time

## Data de‐identification

In Australia, when data are de‐identified, it is legally no longer considered personal information.^27^, ^28^ Data are considered de‐identified when the risk of a person being re‐identified in the data is very low within its data access environment. This means that whether data are considered personal or de‐identified can vary depending on the context in which the data are held.^28^

The process of de‐identifying data involves removal or alteration of personal identifiers, and the application of additional controls or safeguards in the data access environment to appropriately manage the risk of re‐identification.^28^ It is sometimes possible to re‐identify some individuals by interrogating the data with the intention to find individuals by searching for multiple, specific, identifying characteristics of a person who might be represented in the dataset.^29^ Where there is a risk of re‐identification, the data should not be made public. Re‐identification of individuals within public, uncontrolled and purportedly de‐identified datasets has been proven to be possible.^30^ This highlights the importance of the data environment controls and safeguards.

The term “de‐identification” is not consistently defined or used. Other terms used to refer to similar concepts and processes include “anonymisation” and “confidentialisation”.^28^ In the European Union and the UK, “pseudonymisation”, rather than de‐identification, is in common use.

## Data extraction tools and their role

Data extraction tools are software tools designed to extract data from a GP computer system and transmit the data elsewhere for audit, surveillance, data linkage and/or research. Several such tools are in use in Australia – for example, the Canning Tool, cdmNet, GRHANITE, Pen CAT and POLAR GP. Some of these tools have been used to collect primary care data for research for over 10 years, mostly on a resource‐intensive, project‐by‐project basis. Such tools exhibit a variety of features:

- de‐identification of data on extraction and before transmission
- an ability to interface with multiple software systems
- an ability to manage consent
- generation of data linkage keys
- secure data transmission
- facilitation of review of data input quality by practices.

Among these tools, some address data privacy concerns through data being de‐identified or aggregated before they leave the GP clinic. Use of such tools can contribute to research being conducted according to best practice principles (Box 2). However, it is important that other principles — such as independent data governance and ethical, not‐for‐profit use of collected data — are also observed to avoid public backlash against use of de‐identified data. Provisions that manage patients’ consent preferences at the practice, and multiple layers of security to protect data during transmission, are other best practice principles.^17^, ^24^

Privacy‐protecting record linkage enables researchers to examine patients’ journeys through the health care system. It can be enabled by middleware, which generates a unique person‐identifying signature or code. Some types of middleware do this through irreversible “cryptographic hashing” of person‐identifying information while the data are confined still in the clinical setting, so no person‐identifying information is transferred during data transmission.^31^ Others use statistical linkage key (SLK) algorithms (eg, SLK581) to generate signatures from person‐identifying information, but these signatures contain personal data (which are often encrypted).^32^ Approaches like hashing, where no identifiable data are transmitted, are preferable. When data from hospital or administrative datasets are extracted using particular data extraction tools, the same SLKs or hashes may be generated, enabling data linkage. Data linkage has been described as leading to “joined‐up thinking” which, as well as enabling services to better meet public needs, can provide greater perspectives towards finding solutions to intractable problems.^33^

As new technologies allow, additional functions will likely be incorporated into health care‐related data extraction tools, such as collection of consent preferences from patients via apps and smart devices (dynamic consent).^25^ Allowing patients to be personally involved could increase participation rates, trust levels, and the depth and strength of data available for research.^8^, ^25^, ^26^

## Appropriate use of health data for research

The Australian Institute of Health and Welfare suggests that to undertake thorough data‐based research of general practice, data should:

- be analysable at the individual patient level
- be linkable to actions (eg, prescription, clinical procedure, and pathology or imaging request)
- include diagnosis or symptom pattern
- allow tracking of presenting problems and their management over time
- enable examination of patient outcomes.^7^

To use data at the individual level for such purposes, best practice principles must be adhered to (Box 2). We propose a model for using primary care health data for research (Box 3).

**3 A model for primary care data sharing for research**

**1. Preparing for data collection**

- Obtain ethics approval for data collection and undertake legal review.
- Establish a robust and secure data housing environment with independent data governance oversight and proactive security review.
- Establish a comprehensive standard operating procedure and policies for data curation and stewardship.

**2. Recruiting a general practice**

- Establish a legal agreement with the practice and gain their informed consent. This ensures that both parties have a clear understanding of the terms under which data are shared.
- Support any technical requirements for data extraction.
- Inform patients that the practice is sharing de‐identified data. Explicit patient consent is not required if the data extraction tool can provide de‐identified data that satisfies the definition of de‐identification as per the *Privacy Act 1988* (Cth). NHMRC guidelines on waiving patient consent should also be met.^34^ A best practice approach would enable patients to easily withdraw consent.

**3. De‐identifying and transmitting patient and practitioner data**

- Data should be de‐identified on the practice computer.
- Data should be transmitted securely to a protected database in a secure, on‐shore data storage facility.

**4. Following due process**

- Maintain ongoing, proactive data security. This may include using accredited secure environments from which authorised researchers can access the data (depending on sensitivity of the data and the amount of data).
- Ensure that researchers who are provided with data obtain ethics approval and sign a legal agreement stipulating the terms under which they manage, store, use and dispose of the data.
- Use mechanisms to assess competence of researchers to safely and responsibly use the data for research.
- Ensure that the research group includes (or consults with) someone who has experience practising in Australian general practice to ensure that results are interpreted appropriately.
- Ensure that an independent data governance committee reviews all applications by researchers to access data.
- Use principles of data minimisation to limit data sharing with researchers to the minimum necessary to complete their research.

**5. Delivering research outputs**

- Research funders should not prevent researchers from publishing their findings.
- Researchers should make publicly available plain language community reports of their research outcomes.
- Researchers should contribute their data coding to repository‐specific data user groups.

**6. Using consumer, clinician and researcher panels**

- Consult health care consumers and providers — ask them for ideas on how data are used and suggestions regarding potential research projects and questions. Such input should be fed back to researchers to inform future research.
- Engage researchers to contribute insights, data cleaning and analytic codes, so that other research can build on what has already been done.

For GPs and the public to trust any model of data sharing, and consent to data sharing, transparency should be maintained at every stage. Also, the model must adhere to national and international laws, and best practice principles relating to data governance, security, privacy protection and ethical use of data.

In addition to governance issues, capture of poor quality patient data (eg, due to shortcomings in system use such as free text data entry instead of coded data entry) is a limitation of research based on passive capture of EMRs. So improving the quality of data entered in EMRs by GPs is an ongoing aim.^7^, ^14^ Nonetheless, research methods can often adjust for poor quality data capture, so long as the data limitations are clearly understood. Data custodians can help to increase awareness among all clinical and non‐clinical general practice staff of the value of accurate and comprehensive data capture.^7^, ^35^ Having clinician researchers involved in data analysis is one way to ensure correct interpretation of the data. Clinician involvement through research discussion panels can also drive data quality improvements when GPs discover firsthand the implications of poor quality data capture.

Some data extraction tools and primary care data repositories already facilitate timely access to data to generate new knowledge to inform evidence‐based policies, practices and reforms that may translate into cost savings, improved care and better outcomes for patients. Examples of data repositories that do this include NPS MedicineWise's MedicineInsight (www.nps.org.au/medicine-insight) and the University of Melbourne's Data for Decisions research initiative (www.gp.unimelb.edu.au/datafordecisions). In the coming years, use of My Health Record data for research is also likely to increase and should contribute towards these goals.^8^ Policy makers and decision makers need to further support data sharing by providing greater incentives to GPs to contribute data for research, and by addressing jurisdictional barriers and disciplinary silos to enable linkage of datasets.

## Conclusion

Despite most Australians having most of their health‐related interactions in the primary care sector, primary care‐based research is disproportionately low. Access to quality EMR data, lack of resources to remunerate GPs, and a lack of understanding among some GPs of the value and importance of secondary use of EMR data are barriers to data sharing. Data extraction tools that enable ethical, secure and privacy‐protected access to routinely collected datasets nationally have been developed. The task now is to build trustworthy primary care data repositories for research that will provide researchers with timely access to quality‐assured general practice data. Linkage with other datasets could enable significant scale‐up of primary care‐based research in Australia, contributing new knowledge in public health, health promotion, economics and evidence‐based clinical care. Technologies that allow consumers to have greater control over how their data are used can provide better options to policy makers, hence investment in this area is essential. Educating clinicians and the public about the need for, and existence of, research based on de‐identified patient medical records has the potential to generate greater social licence and acceptance of this emerging area of study. This has the potential to generate significant gains in terms of service delivery, economics and patient health. We can “do the right thing” now, but we must never become complacent.

## Competing interests

All authors except Ken Clarke are associated with the Data for Decisions research initiative at the University of Melbourne. Douglas Boyle created the GRHANITE data extraction tool. Douglas Boyle, Joe‐Anne Manski‐Nankervis, Jane Hocking, Jane Gunn and Jon Emery have been investigators on GRHANITE‐related research projects, including NPS MedicineWise projects and a joint project in conjunction with the Australian Digital Health Agency. Douglas Boyle is a member of the Australian Digital Health Agency My Health Record Benefits Measurement Steering Committee. Jon Emery is a member of the NPS MedicineWise MedicineInsight Data Governance Committee.

## Provenance

Commissioned; externally peer reviewed.
